# Triad3a induces the degradation of early necrosome to limit RipK1-dependent cytokine production and necroptosis

**DOI:** 10.1038/s41419-018-0672-0

**Published:** 2018-05-22

**Authors:** Norah A. Alturki, Scott McComb, Ardeshir Ariana, Dikchha Rijal, Robert G. Korneluk, Shao-Cong Sun, Emad Alnemri, Subash Sad

**Affiliations:** 1 Department of Biochemistry, Microbiology, and Immunology, Faculty of Medicine, University of Ottawa, Ottawa, Canada; 2 Applied Medical science, King Saud University, Riyadh, Saudi Arabia; 3 Human Health and Therapeutics, National Research Council of Canada, Ottawa, Canada; 4 Children’s Hospital of Eastern Ontario Research Institute, Ontario, Canada; 5 Department of Immunology, The University of Texas MD Anderson Cancer Center, Houston, TX USA; 6 Thomas Jefferson University, Philadelphia, PA USA; 7 Centre for Infection, Immunity and Inflammation, University of Ottawa, Ontario, Canada

## Abstract

Understanding the molecular signaling in programmed cell death is vital to a practical understanding of inflammation and immune cell function. Here we identify a previously unrecognized mechanism that functions to downregulate the necrosome, a central signaling complex involved in inflammation and necroptosis. We show that RipK1 associates with RipK3 in an early necrosome, independent of RipK3 phosphorylation and MLKL-induced necroptotic death. We find that formation of the early necrosome activates K48-ubiquitin-dependent proteasomal degradation of RipK1, Caspase-8, and other necrosomal proteins. Our results reveal that the E3-ubiquitin ligase Triad3a promotes this negative feedback loop independently of typical RipK1 ubiquitin editing enzymes, cIAPs, A20, or CYLD. Finally, we show that Triad3a-dependent necrosomal degradation limits necroptosis and production of inflammatory cytokines. These results reveal a new mechanism of shutting off necrosome signaling and may pave the way to new strategies for therapeutic manipulation of inflammatory responses.

## Introduction

Macrophage-like cells are present throughout the body and play a major role in initiating inflammatory responses to control pathogens^1–4^. TLR signaling induces MyD88-dependent and TRIF-dependent signaling, which leads to the production of cytokines and chemokines^5–8^, and recruitment of myeloid cells^9^.

Apoptosis promotes cell death during embryogenesis and in the elimination of self-reactive cells^10,11^. Cell death that occurs during infections proceeds through parallel cell death programs^12^. In contrast to apoptosis, inflammatory cell death results in cell-rupture and release of intracellular contents including cytokines and numerous danger-associated molecular patterns (DAMPs) to the external milieu, which induces systemic amplification of inflammation^10,13,14^. Inflammatory cell death by pathogens is now considered to be a key driver of pathogen virulence^15–19^.

While necrosis was once considered to be an accidental, uncontrolled mode of inflammatory cell death, a pathway of regulated necrosis, called “necroptosis” is now known to be induced by TNF-R or IFN-IR engagement^14,17,20–25^. Necrosome signaling involves RipK1–FADD–Caspase-8 interaction, which leads to the phosphorylation of RipK3^26–28^. Activated RipK3 phosphorylates mixed lineage kinase domain-like protein (MLKL), resulting in trimerization of MLKL and its relocation to the cell membrane, causing membrane rupture and necroptosis^29^. We have revealed a novel mechanism of proteasomal degradation of RipK1 and other interacting proteins by Triad3a. Our results indicate that during early necrosome signaling, Triad3a mediates the degradation of RipK3 interacting proteins to regulate necroptosis and expression of inflammatory cytokines.

## Results

### Necrosome activation leads to the degradation of various interacting proteins

**Fig. 1:**
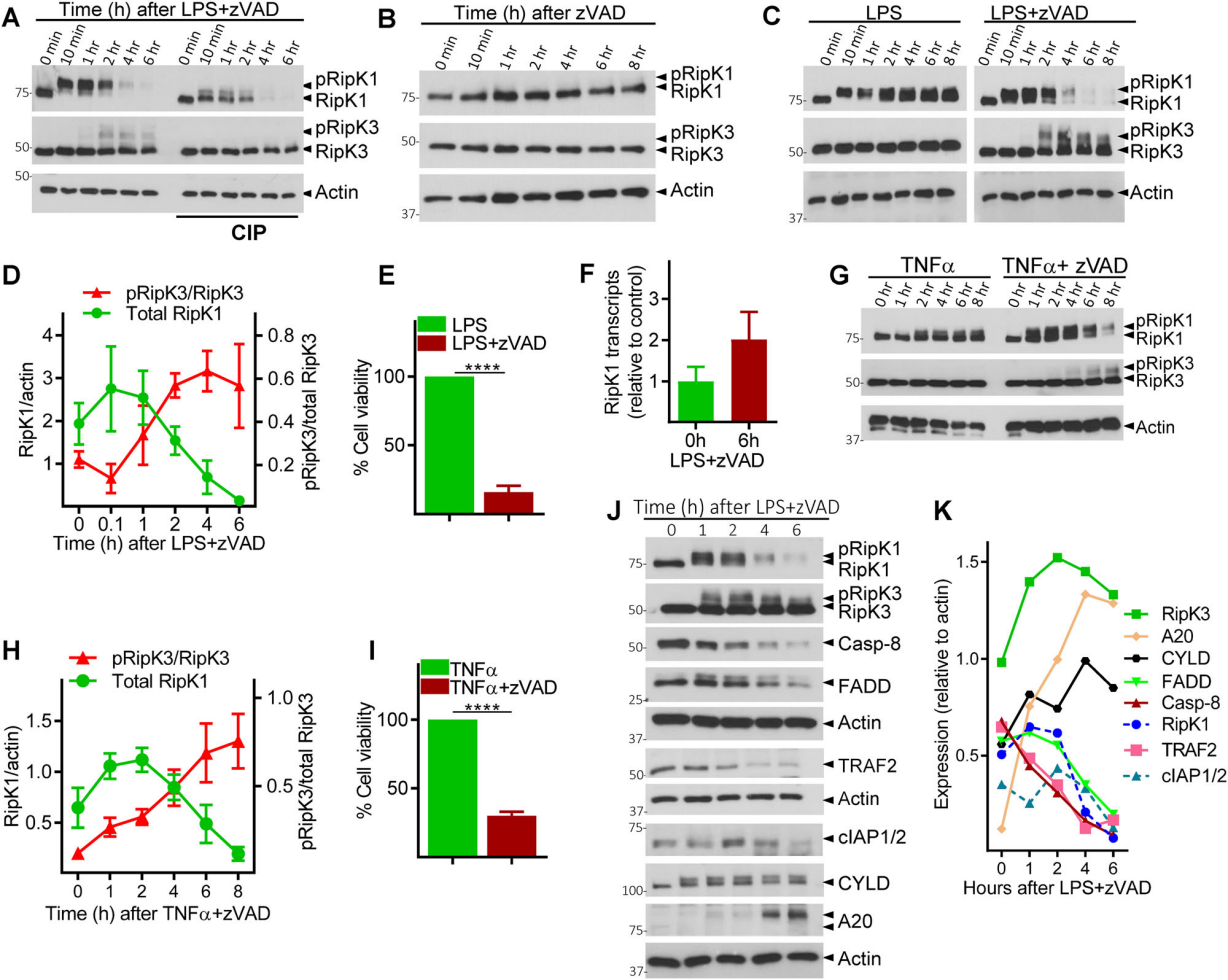
Necrosome signaling leads to degradation of necrosome interacting proteins. **a** Bone marrow-derived macrophages were treated with LPS (100 ng/ml) and zVAD (50 μM). Lysates were collected at various time intervals and examined by western blotting. Phosphatase (CIP, 50 units) was added to some lysates as described in the Methods section. **b**, **c** Macrophages were cultured with zVAD, LPS, or with LPS + zVAD as indicated. Lysates were tested by western blotting. **d** Densitometry analysis of total RipK1 vs. phosphorylated RipK3 in cells treated with LPS + zVAD at various time intervals. **e** Cell viability was measured by MTT assay at 24 h post stimulation of cells with LPS + zVAD. **f** The expression of RipK1 mRNA was examined by quantitative RT-PCR at 6 h post stimulation with LPS + zVAD in comparison to untreated controls. **g** Bone marrow-derived macrophages were treated with TNFα (10 ng/ml) and zVAD (50 μM), and lysates were collected at various time intervals and examined by western blotting. **h** Densitometry analysis of total RipK1 vs. phosphorylated RipK3 in cells treated with TNFα + zVAD at various time intervals. **i** Cell death of macrophages treated with TNFα or TNFα + zVAD was measured by MTT assay at 24 h post stimulation. **j** Macrophages were treated with LPS + zVAD and the expression of various proteins was evaluated by western blotting of cell extracts collected at various time intervals. **k** Densitometric analysis of proteins shown in panel (**j**) was performed. Graphs show the percentage of viable cells ±SEM relative to controls. Each experiment was performed in triplicate and repeated three times. ****P < 0.0001

Combined TLR4 stimulation (by LPS) and caspase inhibition (by zVAD) in macrophages causes the phosphorylation of RipK1 and RipK3, which can be observed as a slightly slower migrating protein band in western blot. Consistent with this, RipK1 and RipK3 were resolved as a single band upon dephosphorylation by calf intestinal phosphatase (CIP) (Fig. 1a). Coincident with phosphorylation of RipK3, we noted a progressive reduction in the levels of RipK1 after 2 h post stimulation (Fig. 1a). Treatment of macrophages with zVAD in the absence of any additional stimulation did not induce the phosphorylation of RipK1 or RipK3, and failed to have any impact on the levels of RipK1 (Fig. 1b). Stimulation of cells with LPS in the absence of zVAD, induced the phosphorylation of RipK1, but not RipK3, without having any impact on the levels of RipK1 (Fig. 1c). Thus, consistent with previous results^30^, combined LPS and zVAD was required for the phosphorylation of RipK3 that correlated with progressive disappearance of RipK1 and eventual cell death by necroptosis (Fig. 1c–e and Fig. S1 A, B). We also measured the expression of protein phosphatase-2, B subunit, a ubiquitous phosphatase in eukaryotic cells, and observed that this protein is also not degraded necrosome signaling (Fig. S1 C). Progressive loss of RipK1 was also observed when cells were stimulated with zVAD in the presence of highly reduced amount of LPS (Fig. S1 D). Reduction in the levels of RipK1 following necroptotic stimulus was not due to poor transcription of RipK1 as measured by qRT-PCR (Fig. 1f).

Although the reduction in RipK1 levels correlated with phosphorylation of RipK3 (Fig. 1d), the relative levels of RipK3 did not diminish during early necrosome signaling, and no cleavage bands of RipK1 or RipK3 were observed (Fig. S1 A, B). Loss of RipK1 was also noted when necroptosis was induced with TNFα + zVAD (Fig. 1g–i), and again correlated with the timing of RipK3 phosphorylation (Fig. 1g, h). Many other components of the necrosome were degraded following LPS + zVAD stimulation, including Caspase-8 (Casp-8), FADD, TRAF2, and cIAP-1 and -2 (Fig. 1j, k). In contrast, there was an increase in the expression of RipK3, CYLD, and A20 following stimulation of cells with LPS + zVAD (Fig. 1j, k), indicating that all proteins are not degraded during necrosome signaling.

Reduction in the levels of Casp-8 following necroptotic stimulus was not due to poor transcription of Casp-8 (Fig. S1 E). Substitution of the pan-caspase inhibitor (zVAD) with the Casp-8 inhibitor (zIETD) failed to induce the loss of RipK1 expression or necroptosis of macrophages (Fig. S1 F, G). While Casp-8 inhibitor reduced the Casp-8 activity to baseline levels (Fig. S1 H), it did not rescue the loss of RipK1 expression following stimulation by LPS + zVAD (Fig. S1 I). This suggests that the loss of RipK1 expression is not due to cleavage of RipK1 by Casp-8. Furthermore, Casp-8 itself was degraded (Fig. 1j).

### Necrosomal degradation is driven by RipK3

**Fig. 2:**
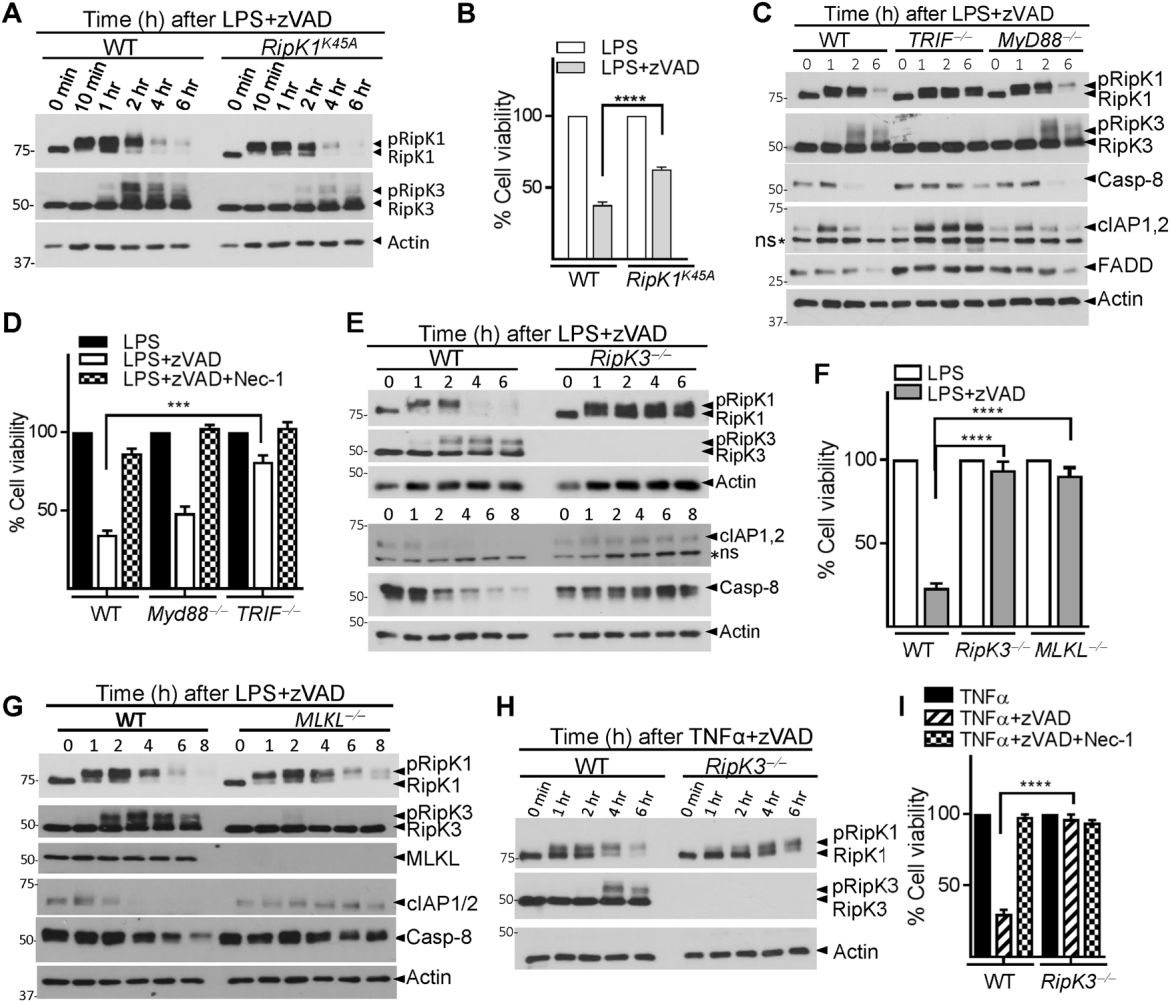
TRIF and RipK3 promotes the degradation of necrosomal proteins. **a** WT and RipK1^K45A^ mutant macrophages were treated with LPS + zVAD. Expression of various proteins was evaluated by western blotting of cell extracts at different time intervals post-stimulation. **b** Cell viability of WT and RipK1^K45A^ macrophages was evaluated by MTT assay at 24 h post stimulation. **c**, **d** WT, TRIF^−/−^, and MyD88^−/−^ macrophages were treated with LPS + zVAD and at various time intervals cell extracts were collected and examined by western blotting (**c**). Cell viability was measured by MTT assay at 24 h post stimulation of cells with LPS + zVAD (**d**). **e**–**g** WT, RipK3^−/−^, and MLKL^−/−^ macrophages were stimulated with LPS + zVAD and extracts were collected at various time intervals and tested for the expression of various proteins indicated in the figure (**e**, **g**). Cell viability was measured by MTT assay at 24 h post stimulation of cells with LPS + zVAD (**f**). **h**, **i** WT and RipK3^−/−^ macrophages were stimulated with TNFα + zVAD and western blot analysis was performed on cell extracts at various time intervals (**h**). Cell viability was evaluated by MTT assay at 24 h post stimulation (**i**). Graphs show the percentage of viable cells ± SEM relative to controls. Each experiment was performed in triplicate and repeated three times. ***P < 0.001, ****P < 0.0001

The canonical kinase function of RipK1 is mediated by the catalytic triad that comprises K45-E63-D156^31^. The two critical kinase regions, K45 and D138 complement each other in driving necrosome signaling and inflammatory responses^32,33^. We next tested whether the K45 kinase region of RipK1, which has been shown to promote necroptosis under some conditions^19^, was required for the degradation of RipK1. K45A mutation of RipK1 resulted in slightly reduced RipK3 phosphorylation and necroptosis but failed to have any significant impact on the degradation of RipK1 (Fig. 2a, b). In contrast, the degradation of RipK1 and necroptosis was dependent on the K45 region of RipK1 when cells were stimulated with TNFα + zVAD (Fig. S2 A, B). Following LPS/zVAD-induced necroptosis, the reduction in the levels of RipK1, Casp-8, cIAP1,2, and FADD was dependent on TRIF, but not on Myd88 signaling (Fig. 2c, d), whereas for TNFα + zVAD stimulation, degradation of RipK1 and necroptosis were not dependent on TRIF (Fig. S2 C, D). Overall, we observed a strong correlation between RipK1 degradation, RipK3 phosphorylation, and eventual progression to necroptotic cell death, and a significant difference in the mechanism of RipK1 degradation following TNF-α- or LPS-induced necrosome signaling.

RipK3-deficient cells were protected against necroptosis and there was no degradation of RipK1, cIAP1/2, and Caspase-8 (Fig. 2e, f and Fig. S2 E). In contrast, when cells were stimulated with LPS only, which does not induced necroptosis, RipK3 did not have any impact on the expression of RipK1, cIAP1/2, and Casp-8 (Fig. S2 F). We wondered about a possible role for the downstream mediator of necroptotic cell death, MLKL^29^. Degradation of RipK1 and Casp-8 was not inhibited in MLKL-deficient macrophages (Fig. 2g and Fig. S2 G). As expected, MLKL- deficient macrophages were resistant to necroptotic cell death (Fig. 2f). We evaluated the expression of these proteins in *Irf9*^*−/−*^ and *Ifnar1*^*−/−*^ macrophages that are resistant to necroptosis^30^. Our results indicate that the degradation of these proteins was not modulated in *Irf9*^*−/−*^ and *Ifnar1*^*−/−*^ macrophages (Fig. S2 H, I), suggesting that degradation continues during necrosome signaling in the absence of cell death. Loss of MLKL led to a significant decrease in RipK3 phosphorylation, suggesting the presence of a feedback role for MLKL in RipK3 phosphorylation (Fig. 2g). RipK3 was also required for necroptosis and the degradation of RipK1 following stimulation of cells with TNFα + zVAD (Fig. 2h, i). Thus, RipK3, but not MLKL is required for necrosomal degradation in macrophages following necroptotic stimulus.

### Necrosomal degradation is driven by early RipK1–RipK3 interaction

**Fig. 3:**
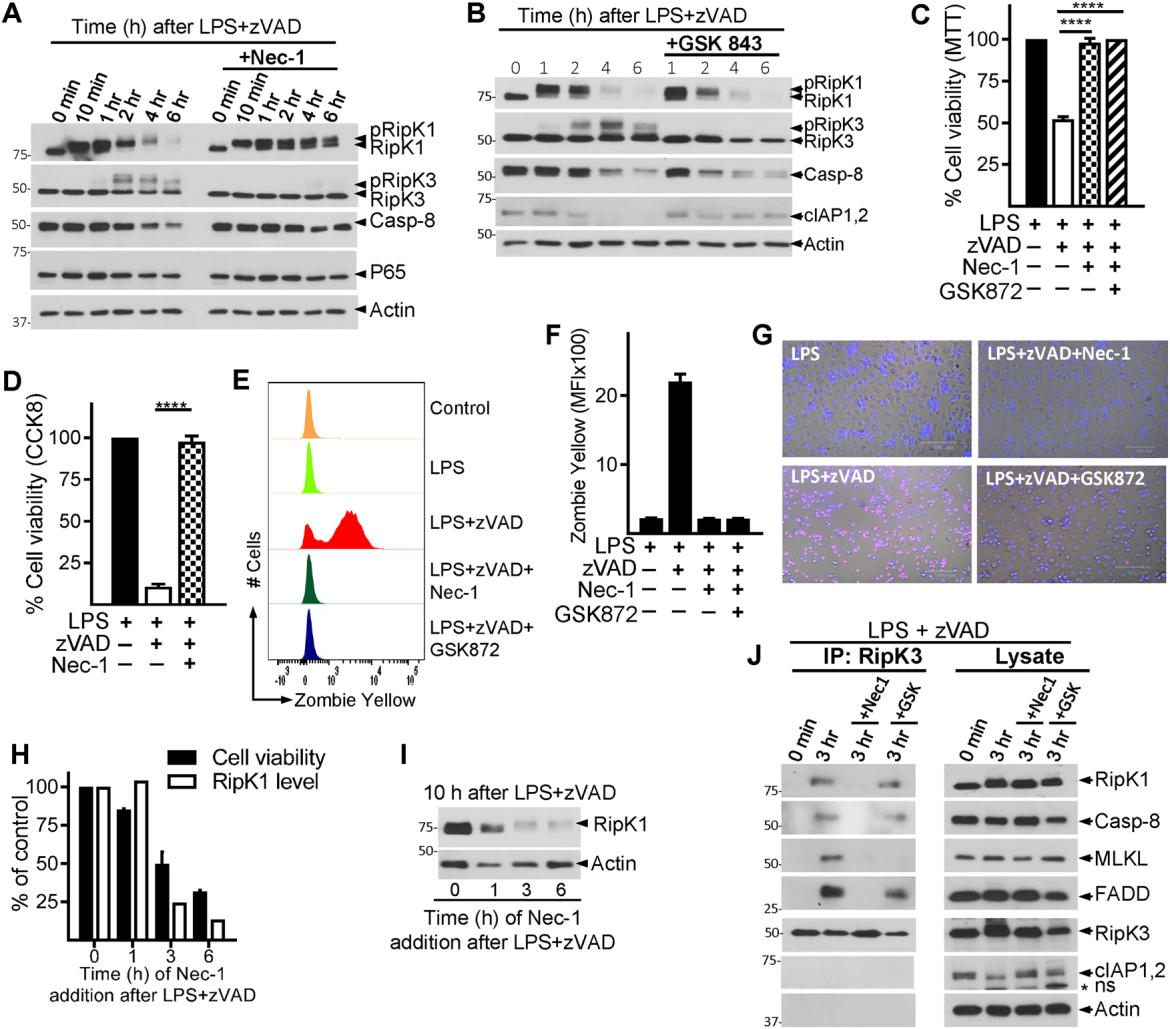
RipK1–RipK3 interaction promotes the degradation of RipK1. **a**–**d** WT macrophages were stimulated with LPS + zVAD in the presence or absence of Nec-1 (30 μM) (**a**, **c**, **d**) or GSK843/872 (3 μM) (**b**). Cell lysates were collected at varying time intervals and examined by western blotting (**a**, **b**). Cell viability of macrophages was examined by MTT (**c**), CCK8 (**d**) assay, Zombie yellow staining (**e**, **f**), and at 24 h post stimulation, or by staining cells with PI/Hoechst (**g**) at 6 h post stimulation. **h**, **i** Macrophages were treated with LPS + zVAD. Nec-1 was added immediately, or at various time intervals post stimulation with LPS + zVAD. Cell viability (**h**) was measured at 24 h by MTT assay, and expression of RipK1 (**i**) was measured by western blotting of extracts collected at 10 h post stimulation with LPS + zVAD. **j** RipK3 was immune-precipitated in control cells and in cells stimulated with LPS + zVAD for 3 h in the absence or presence of Nec-1 or GSK872. Expression of various proteins in the immune-precipitates and cell lysates were examined by western blotting. Graphs show the percentage of viable cells ±SEM relative to controls. Each experiment was performed in triplicate and repeated three times. ****P < 0.0001

Nec-1 has been shown to bind the kinase domain of RipK1 between the N- and C-lobes near the activation loop^34^, and lock RipK1 in an inactive conformation^34^. Nec-1 completely abrogated the phosphorylation of RipK3, degradation of RipK1 and cell death by necroptosis (Fig. 3a, c, d). Similar results were obtained with Nec-1S (Fig. S3 A, B), a more specific inhibitor of RipK1^35^. Nec-1 had no impact on the phosphorylation of RipK1 (Fig. 3a). Inhibition of the p38MAPK resulted in potent inhibition of RipK1 phosphorylation but had no impact on the reduction of RipK1 levels (Fig. S3 C). Despite an obvious decrease in RipK1 phosphorylation by p38MAPK inhibition, this did not affect RipK1 degradation, and resulted in only partial reduction in RipK3 phosphorylation (Fig. S3 C, D), which did not impact necroptosis (Fig. S3 E). Inhibition of p38MAPK significantly reduced TNFα production (Fig S3 F). These results indicate that the phosphorylation of RipK1 itself is not required for the degradation of RipK1.

RipK3-inhibitor GSK843^36^ that completely blocked the phosphorylation of RipK3 (Fig. 3b) and necroptosis (Fig. 3c–g), did not prevent the loss of RipK1 or Casp-8 (Fig. 3b). RipK1 inhibitor (Nec-1) and RipK3 inhibitor (GSK872) showed similar inhibition of necroptosis as measured by MTT (Fig. 3c), CCK8 (Fig. 3d) assay, Zombie yellow staining for dead cells (Fig. 3e, f) and PI/Hoechst staining (Fig. 3g). Interestingly, GSK843 partially restored the levels of cIAP1/2 (Fig. 3b), similar to what was observed with MLKL-deficient cells (Fig. 2g). Similar results were observed with another RipK3 inhibitor (GSK872), which resulted in rescue of cIAP1/2, but not Casp-8 or RipK1 levels (Fig. S3 G, H).

Nec-1, when added at 3 h post-LPS/zVAD treatment was unable to block RipK1 degradation or death (Fig. 3h, i). We performed co-immunoprecipitation with an anti-RipK3 antibody and evaluated the impact of inhibition by Nec-1 vs. GSK872 (Fig. 3j). Addition of Nec-1 completely blocked the association of RipK1, Casp-8, FADD, and MLKL with RipK3 in the necrosome. In contrast, addition of the RipK3-specific inhibitor (GSK872) blocked only the RipK3–MLKL interaction (Fig. 3j). Thus, RipK1–RipK3, but not RipK3–MLKL interaction is necessary for necrosomal degradation. Overall the results suggest the existence of two stages of necrosome signaling, an early necrosome consisting of RipK1, RipK3, Casp-8, FADD (and possibly other components), and a late necrosome complex that includes MLKL and is dependent on RipK3-kinase activity.

### Degradation of necrosome impairs cell death and cytokine expression

**Fig. 4:**
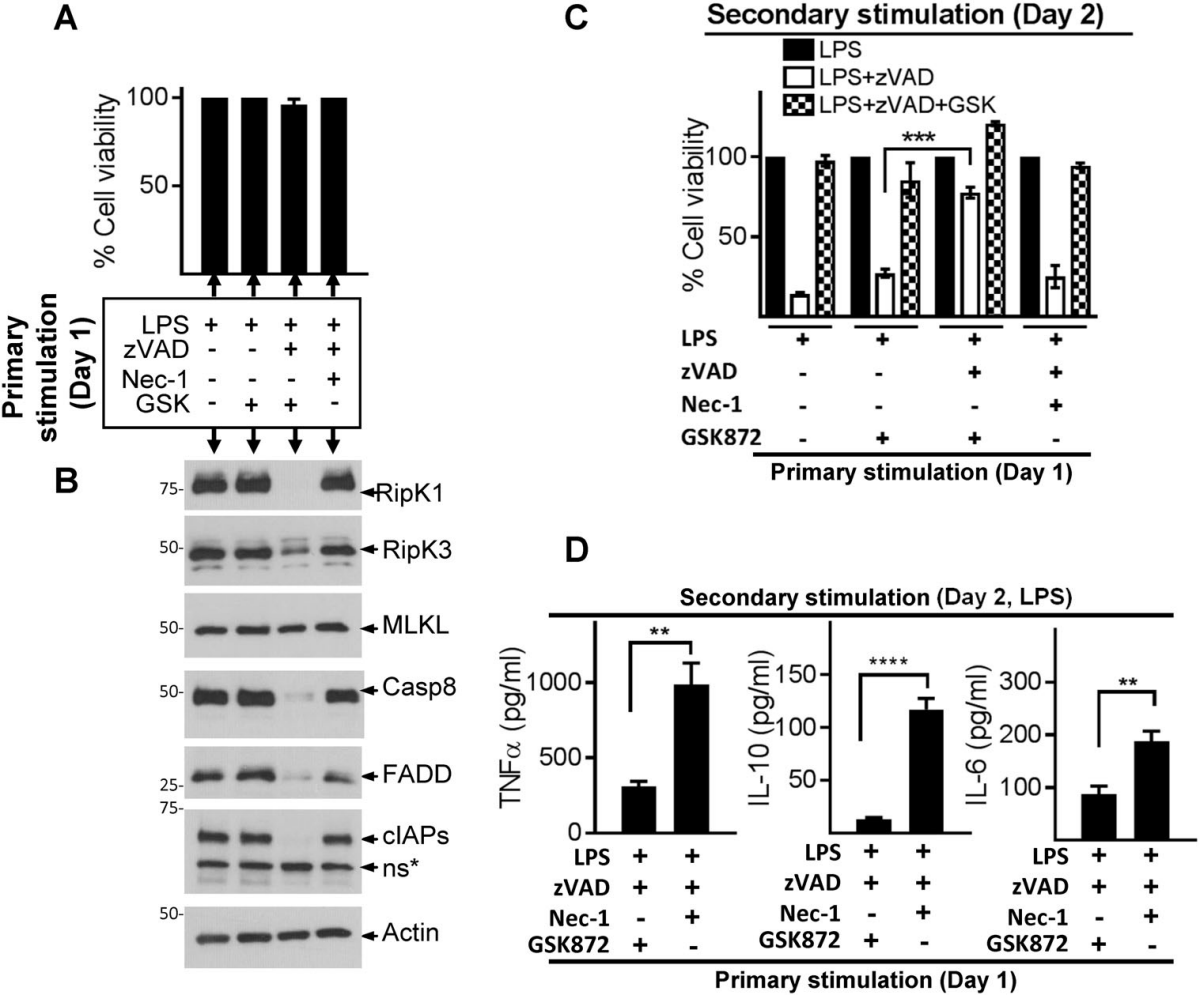
Necrosome degradation results in resistance to cell death and impaired cytokine expression. **a**–**d** WT macrophages were stimulated as described in panel **a**. Cell death was evaluated by MTT at 24 h post stimulation (**a**), and western blotting was performed in cell extracts collected at 24 h (**b**). After primary stimulation (24 h), cells were washed and stimulated with LPS, LPS + zVAD, or LPS + zVAD + GSK872, and cell death was evaluated after another 24 h by MTT assay (**c**). Supernatants were collected at 6 h post secondary stimulation with LPS and expression of cytokines was measured by ELISA (**d**). Graphs show the percentage of viable cells ± SEM relative to cells treated with LPS in the absence of zVAD. Each experiment was repeated thrice with triplicate samples. *P < 0.05, **P < 0.01, ***P < 0.001, ****P < 0.0001

We treated cells with LPS + zVAD to induce necrosome signaling, but blocked cell death using Nec-1 or GSK872. Since Nec-1 and GSK872 have opposite impact on necrosome degradation (Fig. 3a, b), we could specifically disentangle the impact of necrosome degradation on susceptibility to necroptosis and impact on cytokine expression. Primary stimulation of macrophages for 24 h with LPS + zVAD and Nec-1 or GSK resulted in resistance to cell death as expected (Fig. 4a), although only treatment with RipK3 inhibitor (GSK872) resulted in a sustained loss of RipK1, Casp-8, cIAP1/2, and FADD expression (Fig. 4a, b).

We were intrigued to test the properties of macrophages with depleted levels of cell death mediators. Cells that expressed poor levels of necrosomal proteins due to previous treatment with LPS + zVAD and RipK3 inhibitor (GSK872) displayed potent resistance to induction of necroptosis (Fig. 4c). In contrast, cells previously treated with LPS, LPS + GSK872, or LPS + zVAD + Nec-1, which expressed high levels of necrosomal proteins were highly susceptible to necroptotic cell death (Fig. 4c). Degradation of necrosome during primary stimulation (LPS + zVAD + GSK872) also resulted in significantly reduced expression of cytokines in response to secondary stimulation (Fig. 4d). These results reveal that early degradation of the necrosome is a mechanism of cellular escape from necroptosis and cytokine expression.

### RipK3 interaction promotes K48-ubiquitination of necrosome components

**Fig. 5:**
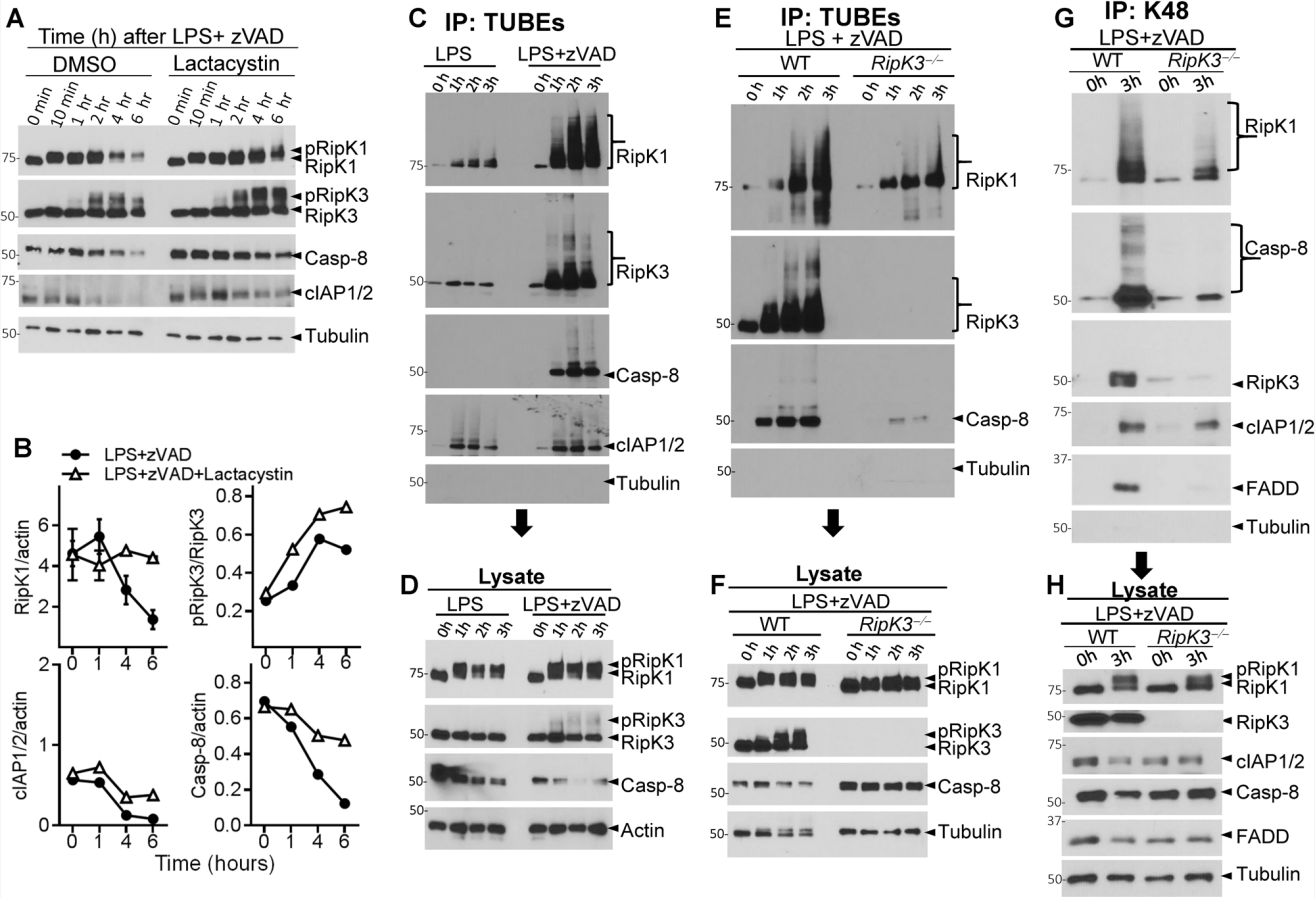
RipK1 is degraded through a proteosomal mechanism. **a** Macrophages were treated with LPS (100 ng/ml) + zVAD (50 μM) in the presence or absence of Lactacystin (10 μg/ml). At various time intervals, cell lysates were collected and examined by western blotting. **b** Densitometric analysis of RipK1, pRipK3, Caspase-8, and cIAP1/2. **c**, **d** WT macrophages were treated with MG132 (10 μM) and LPS (100 ng/ml) or LPS + zVAD (50 μM) for varying time intervals. Lysates were collected and incubated with TUBEs for 16 h at 4 °C as described in Methods. Immuno-precipitates (**c**) and the corresponding lysates (**d**) were examined by western blotting for ubiquitinated proteins. **e**, **f** WT and RipK3^−/−^ macrophages were treated with MG132 (10 μM), LPS (100 ng/ml), and zVAD (50 μM) for varying time intervals as shown. Cells were lysed and immuno-precipitation was then performed using TUBEs as described in Methods. Immuno-precipitates (**e**) and lysates (**f**) were examined by western blotting. **g**, **h** WT and RipK3^−/−^ macrophages were treated with MG132 (10 μM), LPS (100 ng/ml), and zVAD (50 μM) for varying time intervals as shown. Cells were lysed and immuno-precipitation was then performed using magnetic beads coupled with antibodies targeting K-48. Immuno-precipitates (**g**) and lysates (**h**) were examined by western blotting. Each experiment was repeated thrice

We tested whether a proteasome inhibitor, Lactacystin, could rescue the loss of the RipK1/Casp-8 levels. Indeed, Lactacystin induced substantial rescue of RipK1 levels (Fig. 5a, b), which corresponded to an increase in the phosphorylation of RipK3 (Fig. 5a, b). Caspase-8 and cIAP1/2 were partially rescued by Lactacystin treatment (Fig. 5a, b). Similar results were obtained with another proteasome inhibitor, MG341 (Fig. S4 A). Inhibition of transcription (Actinomycin D), endosomal acidification (Chloroquine) and autophagy (hydroxychloroquine) did not rescue the levels of RipK1 (Fig. S4 B).

We immunoprecipitated ubiquitinated proteins with “tandem ubiquitination binding entities” (TUBEs) and performed western blotting of immune-precipitates. We observed that RipK1 was profoundly ubiquitinated following stimulation by LPS + zVAD, but not by LPS (Fig. 5c, d). Similar results were observed when cells were stimulated with TNFα + zVAD (Fig. S4 C, D). Ubiquitination of RipK1 following LPS + zVAD stimulation was significantly reduced in RipK3-deficient macrophages (Fig. 5e, f). Using K48-ubiquitin specific antibody to immuno-precipitate proteins, we observed extensive K48 ubiquitination of RipK1 and Caspase-8 following necrosome activation, which was highly reduced in RipK3-deficient macrophages (Fig. 5g, h). These results show that association of RipK1 and RipK3 in the early necrosome leads to ubiquitination of necrosome components and drives degradation of interacting proteins.

### IAPs do not promote the maintenance of RipK1 during necrosome signaling

**Fig. 6:**
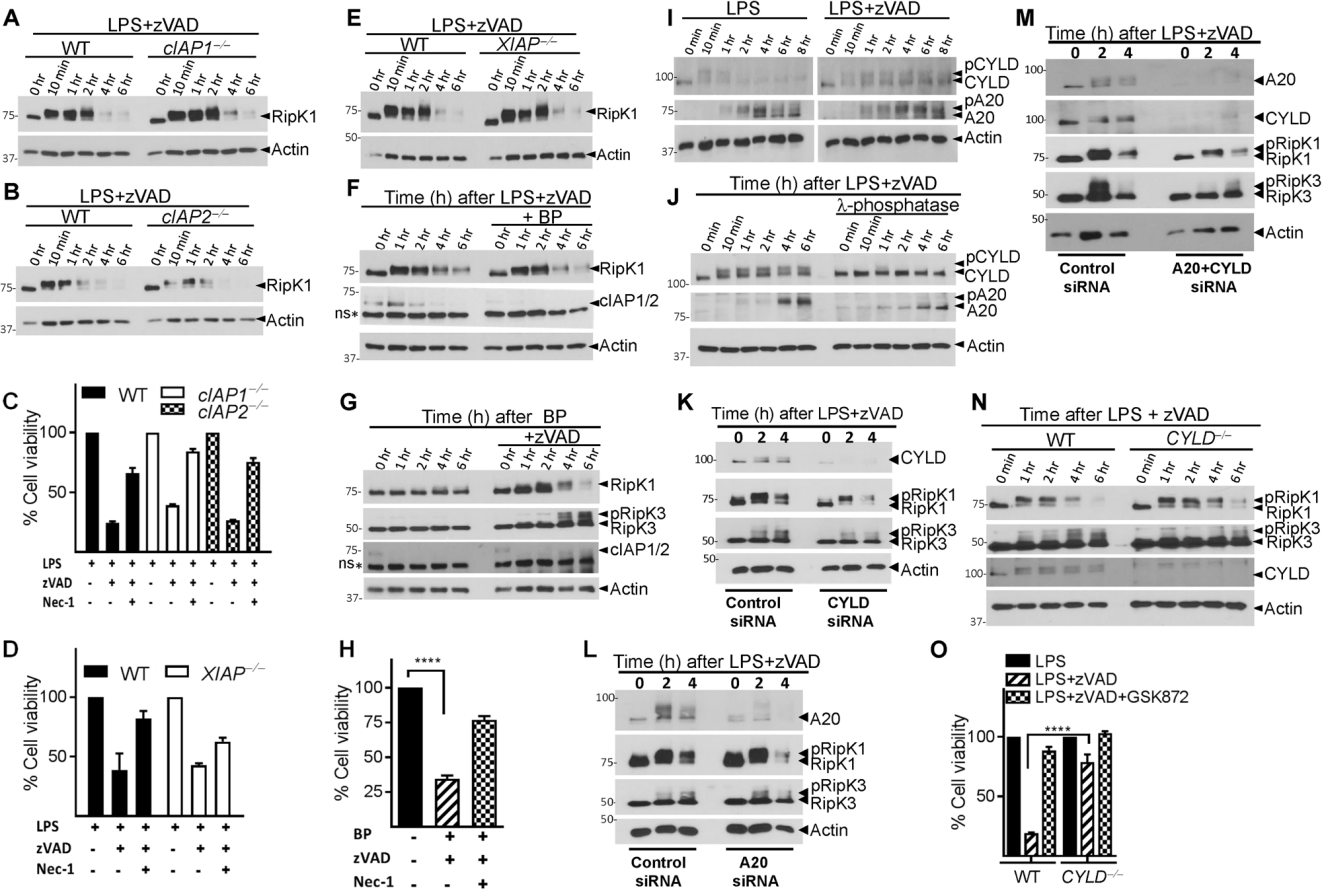
cIAPs, CYLD, and A20 do not mediate the degradation of RipK1. **a**–**e** Macrophages from WT, *cIAP1*^−/−^, *cIAP2*^−/−^, and *XIAP*^−/−^ mice were treated for varying time intervals with LPS + zVAD. Lysates were collected and RipK1 expression was examined by western blotting (**a**, **b**, **e**). Cell viability of WT, *cIAP1*^−/−^*, cIAP2*^−/−^, and *XIAP*^−/−^ macrophages stimulated with LPS + zVAD was measured at 24 h by MTT assay (**c**, **d**). WT macrophages were stimulated with LPS + zVAD in the presence or absence of the SMAC mimetic BP (10 μM), and the expression of RipK1 and cIAP1/2 was measured by western blotting of cell extracts at various time intervals (**f**). Necrosome signaling was induced in WT macrophages by treatment with BP + zVAD, and the expression of RipK1, RipK3, and cIAP1/2 was evaluated by western blotting of cell extracts at various time intervals (**g**). Cell viability was evaluated by MTT assay at 24 h post stimulation with BP + zVAD (**h**). WT macrophages were stimulated with LPS or LPS + zVAD and extracts were collected at various time intervals and tested for the expression of A20 and CYLD (**i**, **j**). Lambda phosphatase (500 units) was added to some lysates as described in the Methods section before performing western blotting (**j**). WT macrophages were transfected with siRNA against CYLD (15 pmol) (**k**) or A20 (15 pmol) (**l**) for 24 h. Cells were then treated with LPS + zVAD for different time intervals and lysates were collected to measure RipK1, RipK3, CYLD, and A20 expression by western blotting (**k**, **l**). WT macrophages were also transfected with siRNA against both CYLD and A20 for 24 h followed by evaluation of the impact on RipK1, RipK3, A20, CYLD expression at various time intervals following LPS + zVAD treatment (**m**). Macrophages from WT, *CYLD*^−/−^, mice were stimulated with LPS + zVAD for various time intervals (**n**, **o**). Expression of RipK1, RipK3, and CYLD was evaluated by performing western blotting of cell extracts (**n**). Cell viability was measured at 24 h post stimulation by MTT assay (**o**). Graphs show the percentage of viable cells ±SEM relative to controls. Each experiment was repeated three times. ****P < 0.0001

Since cIAPs promote K63-ubiquitination of RipK1^37^, we evaluated the role of cIAPs in necrosome degradation. The loss of RipK1 during necrosome signaling remained unperturbed in cIAP1- or cIAP2-deficient macrophages (Fig. 6a, b). Necroptosis of macrophages was also not significantly modulated in cIAP1/2-deficient cells (Fig. 6c). Similarly, the loss of RipK1 or necroptosis was also not influenced by the deficiency of XIAP (Fig. 6d, e). We also pre-treated macrophages for 1 h with the SMAC mimetic Birinapant (BP) which induces rapid degradation of both cIAPs^38,39^. Treatment of macrophages with BP followed by LPS + zVAD resulted in loss of both cIAPs but did not affect the degradation of RipK1 (Fig. 6f). Induction of necroptosis by BP + zVAD treatment also resulted in degradation of RipK1 (Fig. 6g, h).

### A20 and CYLD do not impact the degradation of RipK1 during necrosome signaling

In fibroblasts CYLD and A20 have been shown to edit the ubiquitination of RipK1^40,41^. We observed that the expression of CYLD was increased during necrosome signaling (Figs. 1k and 6i, and Fig. S5 A). CYLD and A20 were phosphorylated during necrosome signaling (Fig. 6j). Phosphorylation of CYLD has been reported to inhibit its deubiquitinase (DUB) activity^42–44^. We performed CYLD and A20 knock-down separately or in combination and evaluated the impact on RipK1 degradation. Rather than inhibiting RipK1 degradation, knock-down of CYLD and/or A20 appeared to enhance the degradation of RipK1 (Fig. 6k–m and Fig. S5 C, D), suggesting that CYLD and A20 promote maintenance of RipK1. Similarly, CYLD-deficient macrophages had little change in degradation of RipK1, although phosphorylation of RipK3 and necroptosis was significantly lower relative to WT macrophages (Fig. 6n, o). Inhibition of the linear ubiquitin chain assembly complex (LUBAC) by gliotoxin^45^ also did not have any impact on the degradation of RipK1 or necroptosis (Fig. S5 F, G).

### Triad3a promotes necrosome degradation and regulates cell death and cytokine production

**Fig. 7:**
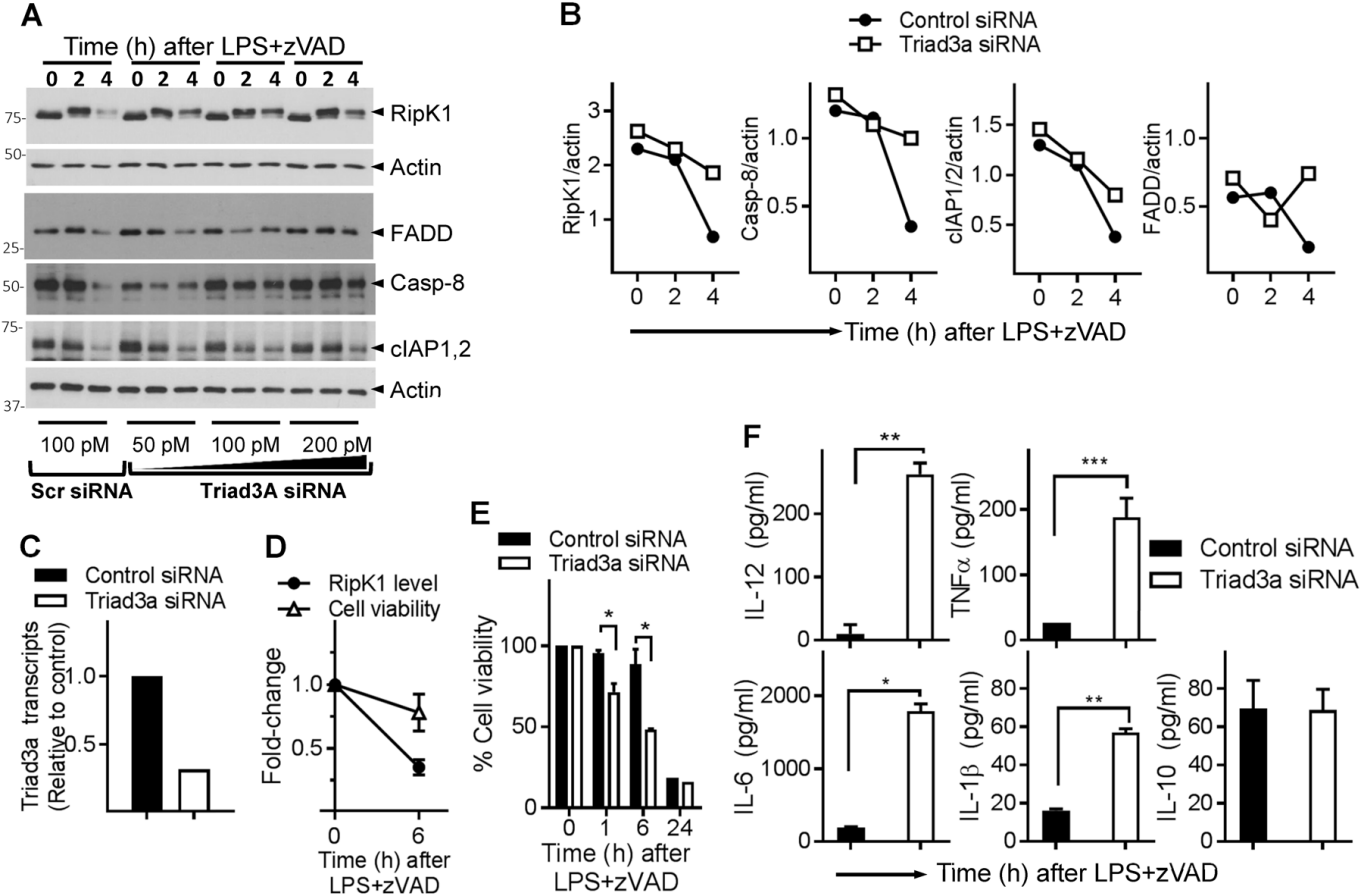
Triad3a promotes the degradation of necrosome and regulates necroptosis and cytokine expression. **a**–**e** WT macrophages were transfected with siRNA against Triad3a (50, 100, or 200 pmol) for 24 h. Control cells were transfected with scrambled siRNA (100 pmol). Cells were then treated with LPS + zVAD for different time intervals and lysates collected for western blotting (**a**). Densitometric analysis of representative western blots transfected with 100 pM siRNA was performed (**b**). Expression of Triad3a was evaluated at 24 h post transfection by qRT-PCR (**c**). WT macrophges were treated with LPS (1 ng/ml) + zVAD (50 μM), and the impact on cell death (MTT assay) and RipK1-expression (western blotting) evaluated (**d**). Primary macrophages were transfected with control or Triad3a siRNA for 24 h, and cells were washed next day and cell death was evaluated at various time intervals post stimulation of cells with LPS (1 ng/ml) + zVAD (50 μM) by MTT assay (**e**). Macrophages were transfected with siRNA against Triad3a as mentioned above. At 24 h, cells were washed and stimulated with LPS (100 ng/ml) + zVAD (50 μM). Cytokines were measured in cell supernatants by ELISA (**f**). Graphs show the percentage of viable cells ±SEM relative to cells treated with LPS in the absence of zVAD. Each experiment was repeated thrice with triplicate samples. *P < 0.05, **P < 0.01

Stimulation of cells with LPS/zVAD initiated cell death without any evidence of RipK1 degradation for the first few hours (Fig. S6 A, B). There was a deceleration in the magnitude of cell death between 3 and 8 h that correlated with a precipitous decrease in necrosomal proteins, suggesting that the loss of necrosomal proteins may act as a regulatory mechanism to limit cell death. Triad3a is an E3 ubiquitin ligase that interacts with TLRs^46^, TRIF/RipK1^47^ and regulates TLR signaling^46^. Since necrosome signaling and degradation was inhibited in *TRIF*^*−/−*^ macrophages (Fig. 2c, d), we evaluated whether Triad3a impacts the degradation of RipK1 during necrosome signaling. Knock-down of Triad3a by siRNA resulted in reduction in the degradation of RipK1, FADD, and Caspase-8 (Fig. 7a–c), indicating that Triad3a promotes the degradation of these proteins during necrosome signaling. Knock-down of Triad3a did not have a significant impact on the degradation of cIAP1/2 (Fig. 7a, b). In the absence of any high-quality western blot antibody that could specifically detect mouse Triad3a, we tested the efficiency of Triad3a knock-down by qRT-PCR, which showed substantial reduction in Triad3a levels (Fig. 7c). Stimulation of cells with LPS + zVAD had a marginal impact on cell death at earlier time periods (Fig. 7d, e), knock-down of Triad3a resulted in slightly enhanced cell death (Fig. 7e). Triad3a knock-down resulted in substantial increase in the expression of inflammatory cytokines following necrosome signaling (Fig. 7f). Overall, these results indicate a model wherein recruitment of RipK3 to the early necrosome leads to Triad3a-dependent K48 ubiquitination of RipK1 and other necosome components as an auto-regulatory mechanism that limits both cell death and inflammatory cytokine production.

## Discussion

Since macrophages promote inflammatory responses in various tissues, and necrosome signaling leads to exacerbation of inflammatory responses^13,48,49^, understanding the mechanisms of regulation of necrosome signaling in macrophages is crucial. The mechanisms through which RipK1 and RipK3 are phosphorylated during necrosome signaling are unclear^14,50^. TLR signaling via TRIF has been shown to promote phosphorylation of RipK1 in macrophages^51^, however, our results indicate that TRIF-deficient macrophages had no impairment in phosphorylation of RipK1. Inhibition of RipK1 by Nec-1^50^ also did not abolish the phosphorylation of RipK1. Furthermore, RipK1^K45A^ kinase-inactive macrophages also had no impairment in phosphorylation of RipK1, supporting a view that RipK1 does not auto-phosphorylate itself. IKK complex has been previously shown to phosphorylate RipK1^52^. MK2, the downstream target of p38-MAPK was shown to mediate an inhibitory phosphorylation on RipK1^53^. These results support a complex model of RipK1 phosphorylation, wherein multiple kinases can phosphorylate RipK1 at multiple sites which can both inhibit and activate necroptotic cell death^52,54^.

Downstream of RipK1, we and others have previously shown that TRIF signaling is necessary for phosphorylation of RipK3 in macrophages^30,51^. Interestingly, here we observe that Nec-1 inhibited the interaction of RipK1 and RipK3, and blocked the phosphorylation of RipK3, leading to inhibition of necroptosis and rescue of degradation of various necrosomal proteins. While previous results have been interpreted to indicate that RipK1 can phosphorylate RipK3, our results support a view that Nec-1 blocks RipK1–RipK3 interaction and does not necessarily impact on kinase function. More recently, convincing evidence has emerged to show that RipK3 dimerization leads to auto-phosphorylation, which is key for downstream activation of MLKL^55,56^. We add here a curious observation that the phosphorylation of RipK3 is abrogated in MLKL-deficient macrophages, suggesting that RipK3-MLKL platform may facilitate RipK1-mediated phosphorylation or auto-phosphorylation of RipK3.

Our results indicate that while the kinase function of RipK3 is necessary to activate necroptosis, interaction with RipK3 leads to the degradation of RipK1 and other necrosomal proteins. This represents a complex model wherein RipK3 appears to have contrasting functions. Our results suggest that the formation of an early necrosome platform that initiates a decision between degradation via K48-ubiquitination or cell death via RipK3 kinase activity and MLKL recruitment.

Several ubiquitin ligases have been shown to ubiquitinate RipK1 in TNF-R complex-I, including cIAPs, TRAF2, and LUBAC. All of these ubiquitinations in the complex I (M1- or K63-ubiquitin) appear to promote RipK1 signaling, not degradation^57^. Consistent with this, our results indicate that treatment of cells with TNFα or LPS alone does not induce RipK1 degradation. Rather, we find that RipK1 degradation only occurs following association with RipK3, and is impacted by Triad3a. Interestingly, Triad3a has been shown to cause degradation and regulation of TLR signaling, including RipK1^46,47^. Due to the lack of availability of a suitable antibody, we have been unable to confirm whether RipK3 directly recruits Triad3a to the necrosome complex.

Various DUBs such as A20 and CYLD remove the ubiquitination chains on RipK1 as the cells transition toward the necrosome signaling platform^58,59^. Our results show that the expression and phosphorylation of CYLD increased during necrosome signaling. Interestingly, phosphorylation of CYLD has been shown to inhibit its DUB activity^42–44^.

Casp-8^60,61^, cIAPs^38^, and Cathepsins^62^ regulate necroptosis in macrophages. Here we have introduced a new mechanism of self-regulation built into necroptotic signaling. Similar to the inactivating timer mechanism identified in the apoptosome^63^, we reveal here that recruitment of RipK3 to the necrosome initiates a competition between necrosomal degradation and necrosomal maturation. This would suggest that the rate of RipK3 kinase activation and MLKL recruitment determine the proportion of necroptotic death that occurs. By applying small molecule inhibitors of RipK3, we were able to prevent activation of the late necrosome, resulting in cells with degraded necrosomal machinery and resistance to subsequent necroptotic challenge.

RipK1^19,33^ and RipK3^13,17,30^ promote inflammatory responses, and it is not clear whether this is related to the role of these proteins outside the necrosome complex^64,65^. In contrast to apoptosis, which is considered to be immunologically silent or immune-suppressive^66^, the disruption of the cellular membrane and release of DAMPs during necroptosis is expected to promote inflammatory responses^67^. Our data indicate that proteasomal degradation of RipK1 and other early necrosome components acts to destabilize death inducing complexes and prevent the completion of the necroptosis program. It is conceivable that this could result in a pool of cells that survive cell death induction and maintain key functionality.

## Methods

### Mice

C57BL6/J (Jax #000664), *TRIF*^*−/−*^ (Jax #005037), *Myd88*^*−/−*^ (Jax #009088), and *TNFR1/2*^*−/−*^ (Jax #003243) were obtained from Jackson Labs (Bar Harbor, USA). *RipK3*^*−/−*^ were a kind gift of Dr. Vishva Dixit (Genentech, San Francisco, CA, USA); RipK1^K45A^ mice and wild type litter mates were a kind gift of Dr. Peter J. Gough (GlaxoSmithKline, Collegeville, USA); *MLKL*^*−/−*^ bone marrow samples were received from Dr. Emad Alnemri (Thomas Jefferson University, Philadelphia, USA). *CYLD*^*−/−*^ mice were as previously described^68^. Experiments were performed in accordance with the Canadian Council on Animal Care guidelines and the Ethics Board and/or the Animal Care Committee at University of Ottawa.

### Reagents

Pan-caspase inhibitor, z-VAD-fmk (#A1902) was obtained from ApexBio (Houston, USA). Lactacystin (L6785), MG132 (C2211), cyclohexamide (C4859), hydroxychloroquine (H0915), and ultrapure LPS (*E. coli* 0111:B4, L3024) were obtained from Sigma-Aldrich (Oakville, Canada). Necroatatin-1 (9037) was obtained from Sigma Chem Co. (Oakville, Canada). M-CSF (416-ML) and TNFα (410-MT) were obtained from R&D (Minneapolis, USA). Birinapant (S7015) was obtained from Selleckchem (Houston, USA). GSK843 and GSK'872 (AOB4898, AOB488) were obtained from Aobious (Gloucester, USA). Caspase-8 Inhibitor, z-IETD-FMK (064-20C) was obtained from BioVision (San Francisco, USA). P38 inhibitor (5633S) was obtained from Cell Signaling (Danvers, USA). Alkaline Phosphatase, Calf Intestinal (CIP) (M0290, NEB). Protease inhibitors (04693132001) were obtained from Roche Applied Science (Laval, Canada). PR-619 (SI9619), 1,10-phenanthroline (SI9649) and GST-tagged tandem ubiquitin-binding entities (TUBE) (UM102) were obtained from LifeSensors (Malvern, USA). Actinomycin-D (0219452505) was obtained from MP Biomedicals (Solon, USA). Necrostatin-1s (cat# 504297) and MG341 (Bortezomib) (cat# 504314) were obtained from Calbiochem (San Diego, CA, USA). Zombie Yellow™ Fixable Viability Kit cat# 423103, Biolegend (San Diego, USA).

### Generation of macrophages

Primary bone marrow-derived macrophages (BMDMs) were differentiated in the presence of 5 ng/ml of M-CSF for 6 days at 37 °C in R8 media (RPMI, 8% fetal bovine serum, 50 µM β-mercaptoethanol and 50 ng/ml gentamycin)^30^.

### Cell culture and viability assay

BMDMs were stimulated in either 24- or 96-well tissue culture plates with LPS (100 ng/ml) or TNF-α (1000 U/ml), in the presence of pan-caspase inhibitor zVAD (50 μM). In some experiments, BMDM were co-treated with various inhibitors and agonists 30 min before stimulation and left for a couple of hours before assaying the cell viability or collecting protein lysate. Cell viability was measured using 3-[4,5-dimethylthiazol-2-yl]-2,5-diphenyltetrazolium bromide (MTT) assay. The MTT reagent was diluted with R8 media at a final concentration 0.5 mg/ml and incubated at 37 °C. After 2 h, 5 mM HCl isopropyl alcohol were added to solubilize MTT crystals and absorbance was measured at a wavelength of 570 nm with a reference wavelength of 650 nm on a Molecular Devices FilterMax plate reader. Cell Counting Kit-8 (CCK-8) was used and 10 μl was added to each well and incubated at 37 °C for 2–4 h. After incubation the absorbance was measured at 450 nm using Molecular Devices FilterMax plate reader. CCK-8 (cat# CK04-01) was obtained from Dojindo Molecular Technologies (Rockville, USA).

### Flow cytometry

Zombie Yellow™ (BioLegend, San Diego, CA) was used to assess cell viability. Using BPS buffer, 24 h after treating the cells in a 96-well plate with various treatments, the seeded cells were washed twice. Without detaching the cells, 50 μl of diluted Zombie Yellow™ solution (1:100 in PBS) was added into each well and the plates were incubated at room temperature, in the dark, for 15–30 min before washing with 100 μl FACS buffer (PBS, 1% BSA, 1 mM EDTA) once. Then, samples were fixed by using 1% paraformaldehyde and analyzed with a LSR Fortessa cell analyzer and FACSDiva software (Becton Dickinson). The data were analyzed with FlowJo software.

### Quantitative RT-PCR

Total RNA was isolated using TRIzol reagent (Life Technologies). Isolated RNA was reverse transcribed into cDNA in a 20-μl reaction volume using SuperScript III Reverse Transcriptase (Invitrogen,18080– 044) as follows: 1 μg of RNA template was added to 0.5 μl of oligo(dT) (50 μM), 0.5 μl of random primers (50–250 ng), 4 μl of 5× First Strand buffer, 1 μl of DTT (0.1 M), 1 μl of dNTP (10 mM), 1 μl of RNase OUT (40 units/μl), and 1 μl of SuperScript III (200 units/μl). After cDNA synthesis, 2 μl of cDNA was analyzed using the SYBR Green (Life Technologies) fast method performed on an Applied Biosystems 7500 quantitative RT-PCR system. The primers used were as follows: Actin (forward) 5′-GATCAAGATCATTGCTCCTCCTG-3′, (reverse) 5′-AGGGTGTAAAACGCAGCTCA-3′; RipK1 (forward) 5′-GGCCAACATTTCTTGGCATTGA-3′, (reverse) 5′-CTGCAGCACTGGGCTTTGAT-3′; Caspase-8 (forward) 5′-TGCCCAGATTTCTCCCTACA-3′, (reverse) 5′-AAGCAGGCTCAAGTCATCTTCC-3′. Triad3a (forward) 5′-ACAGATGATCACCATGTTTGGTT-3′, (reverse) 5′-CATCCATTCCTTTCCCCGGT-3′.

### RNA interference

The smart pool ON-TARGETplus siRNA against CYLD, A20, and TRIAD3A were purchased from Dharmacon (GE Healthcare, USA). BMDMs were transfected with siRNA using Lipofectamine® RNAiMAX Transfection Reagent (13778030, Invitrogen) according to the manufacturer’s instructions. After 24 h, the transfected cells were used for time course experiments. Sequence of the siRNAs is provided in the supplemental section.

### Western blotting

Cells were stimulated as indicated and lysed in SDS lysis buffer with 1% β-mercaptoethanol and boiled immediately at 95 °C for 5 min. Lysates were separated by 10% or 8% SDS-PAGE and transferred to PVDF membrane. Immunoblot analysis was performed using the following antibodies: mouse anti-RipK1 (610458, BD Biosciences, Mississauga, Canada), rabbit anti-RipK3 (2283, ProSci Inc., Poway, USA), mouse anti-actin (47778, Santa Cruz Biotechnology, Dallas, USA), rabbit anti-STAT1 (9172, Cell Signaling), rat anti-caspase-8 (1G12, Enzo), rabbit anti-CIAPs (CY-P1041, Cyclex), rat anti-MLKL monoclonal (MABC604, EMD Millipore), rabbit anti-CYLD (8462, Cell Signaling), rabbit anti-A20 (5630, Cell Signaling, Danvers, USA), mouse anti-FADD (1F7, Enzo, Farmingdale, USA) and mouse anti-αTublin (5286, Santa Cruz Biotechnology, Dallas, USA). Rabbit anti-PP2A B subunit (100C1) was obtained from Cell Signaling Inc. In some cases, whole cell lysates were prepared in RIPA-EDTA free buffer supplemented with protease inhibitor and 50 Units of calf intestinal phosphatase (CIP) was used to dephosphorylate the total protein in 1× CutSmart NEB buffer for 50 min at 37 °C. SDS lysis buffer was added to the treated samples and boiled for 5 min at 95 °C followed by standard western blot protocol.

### Immunoprecipitations

Cell lysates were immunoprecipitated with Dynabeads co-immunoprecipitation kit (Invitrogen, Life Technologies, Burlington, Canada) with the following antibodies: rabbit anti-RipK3 (2283, ProSci Inc., Poway, USA), rabbit anti-K48 ubiquitin (Apu2, Millipore, USA), and rabbit anti-K63 ubiquitin (Apu3, Millipore, Burlington, Canada). Bound proteins were eluted and subsequently analyzed by western blot standard protocol. The densitometric quantification of western blot signals was performed using ImageJ 1.48 software (Maryland, USA).

For immune-precipitation with tandem ubiquitin-binding entities (TUBES), BMDMs were treated with proteasome inhibitor MG132 (5 μg/ml) before stimulation with LPS/zVAD or TNF/zVAD. Cells were washed with ice-cold 1× PB and lysed in buffer contains 50 mM Tris-HCL pH 7.5, 150 mM NaCl, 1 mM EDTA, 1% NP-40, 10% glycerol, protease inhibitor, phosphatase inhibitor, 20 μM deubiquitinase inhibitor PR-619 and 1× 1,10-phenanthroline. The lysates were sonicated for 10 s 3 times and then centrifuged at 14,000 × *g* for 30 min at 4 °C to pellet cell debris. The clarified lysate were then incubated with equilibrated Agarose-TUBEs for 16–24 h at 4 °C on a rocker platform. After incubation, the beads were collected by low speed centrifugation 5000 × *g* for 5 min at 4 °C. The unbound fractions were saved at −20 °C and the beads were washed 3 times with ice-cold TBS-T washing buffer that contains 20 mM Tris-HCL pH 8, 150 mM NaCl and 0.1% Tween-20. The resin was re-suspended in 40 μl of 2× SDS lysis buffer and boiled for 5 min at 95 °C then centrifuged at 13,000 × *g* for 5 min at room temperature. The eluent was analyzed by standard western blot protocol.

### Cytokine analysis

Supernatants were collected from 24-well plates and the expression of cytokines was assessed using the BD optEIA ELISA kit (eBioscience, CA, USA) according to the manufacturer’s instructions. The absorbance was read at 450–570 nm on a FilterMax F5 Multimode microplate reader (Molecular Devices).

### Statistical analyses

All graphs show the average result taken from at least three independent experiments. Error bars show the standard error of the mean, and statistical significance between groups were determined by using Student’s *t*-test using Graphpad Prism 6.07 software package (La Jolla, California, USA).

## Electronic supplementary material

Supplemental figure legends

Supplemental Figure 1

Supplemental Figure 2

Supplemental Figure 3

Supplemental Figure 4

Supplemental Figure 5

Supplemental Figure 6

Supplemental data siRNA sequences
