# Supplementary material for: Triad3a induces the degradation of early necrosome to limit RipK1-dependent cytokine production and necroptosis
Source: Cell Death Dis. 2018 May 22;9(6):592. doi: 10.1038/s41419-018-0672-0 (PMC5964080; doi:10.1038/s41419-018-0672-0)
Supplement: Supplementary file 1 — Supplemental figure legends [file 41419_2018_672_MOESM1_ESM.docx]

**Supplemental figure legends**

**Figure S1.** **Necroptotic stimulation does not lead to cleavage of RipK1.** Western blot analysis of RipK1 and RipK3 expression was performed in macrophages treated with LPS (100ng/ml) and zVAD (50 μM). Expression of RipK1 (A), RipK3 (B) and PP2A B subunit (C) was evaluated as described in experimental methods. (D) Macrophages were stimulated with zVAD (50μM) and 1ng/ml LPS or 100 ng/ml LPS. At various time intervals the expression of RipK1 was evaluated by performing western blotting of cell extracts. (E) WT macrophages were treated with LPS (100ng/ml)+zVAD (50μM) and Caspase-8 expression (mRNA) was evaluated by quantitative RT-PCR at 6 hours. (F-I) WT macrophages were treated with LPS+zVAD, LPS+zIETD, or LPS+zVAD+zIETD. Lysates were collected at various time intervals and the expression of RipK1 evaluated by western blotting. (G) WT macrophages were stimulated as indicated in the panel; LPS (100ng/ml), zVAD (50 μM), zIETD (50 μM) and Nec-1 (30 μM). Cell viability was evaluated at 24h by MTT assay. (H) Macrophages were treated as indicated in the panel and Casp-8 activity evaluated with luciferase kit from Promega at 3 hours post treatment. (I) Macrophages were treated with LPS+zVAD in the absence or presence of Casp-8 inhibitor (zIETD). At various time intervals, extracts were collected for western blotting. Graphs show the percentage of viable cells±SEM relative to cells treated with LPS in the absence of zVAD. Each experiment was repeated thrice with triplicate samples.

**Figure S2. RipK3 promotes degradation of RipK1 during TNFα− and LPS− induced necrosome signaling.** (A, B) WT and RipK1^K45A^ mutant macrophages were treated with TNFα+zVAD, and expression of RipK1 and RipK3 was evaluated by western blotting of cell extracts at various time intervals. (B) Cell viability of WT and RipK1^K45A^ macrophages was evaluated by MTT assay at 24 h post-stimulation. (C, D) WT, TRIF^−/−^ and MyD88^−/−^ macrophages were treated with TNFα+zVAD. Cell extracts were collected at various time intervals and examined by western blotting (C). Cell viability was measured by MTT assay at 24 h post-stimulation of cells with TNFα+zVAD (D). (E) WT and *RipK3^−/−^* macrophages were treated with LPS+zVAD and densitometric analysis was performed on western blots of cell extracts shown in Fig. 2 E. (F) WT and *RipK3^−/−^* macrophages were treated with LPS for various time intervals and the expression of RipK1, cIAP1/2, Ripk3 and Casp-8 was evaluated by western blotting of cell extracts. (G) WT and *MLKL^−/−^* macrophages were treated with LPS+zVAD and densitometric analysis was performed on western blots of cell extracts shown in Fig. 2 G. (H, I) WT, *Irf9^−/−^* and *Ifnar1^−/−^* macrophages were stimulated wit LPS+zVAD as described above and the expression of various proteins evaluated by western blotting. Graphs show the percentage of viable cells ± SEM relative to controls. Each experiment was performed in triplicate and repeated three times. ***P < 0.001, ****P < 0.0001.

**Figure S3. Degradation of RipK1 is not dependent on the phosphorylation of RipK1, and the kinase activity of RipK1/RipK3.** (A, B) Macrophages were treated with LPS+zVAD in the presence or absence of the Nec-1S inhibitor. Expression of various proteins was evaluated by western blotting of cell extracts (A). Cell death was evaluated by MTT assay at 24h post stimulation (B). (C-F) Macrophages were treated with LPS+zVAD in the presence or absence of the p38 MAPK inhibitor (50 μM) for varying time intervals as shown. Lysates then were examined by western blotting (C). Densitometric analysis of representative western blots was performed (D). Cell viability of macrophages treated with LPS+zVAD in the absence or presence of the p38 MAPK inhibitor (50 μM) was evaluated at 24h by MTT assay (E). Expression of TNFα was measured in the supernatant collected at 6h post stimulation (D). (G, H) WT macrophages were treated with LPS+zVAD in the presence or absence of GSK843 or GSK872 (3 μM). Cell lysates were collected at various time intervals and tested for the expression of various proteins. Graphs show the percentage of viable cells±SEM relative to cells treated with LPS in the absence of zVAD. Each experiment was repeated thrice with triplicate samples. *P < 0.05.

**Figure S4. Necrosome is not degraded through a lysosomal mechanism, and necrosome signaling by TNF+zVAD also leads to increased ubiquitination of RipK1.** (A) Macrophages were treated with LPS (100ng/ml)+zVAD (50 μM) in the presence or absence of proteasome inhibitor MG341 (10 μM). Cell lysates were collected and western blotting performed to measure protein expression. (B) Macrophages were treated with LPS (100ng/ml)+zVAD (50 μM) in the presence or absence of Actinomycin-D (80 nM), Cycloheximide (1.7 µM) and Hydroxychloroquine (100 µM). Cell lysates were collected at various time intervals and the expression of RipK1 evaluated by western blotting. (C, D) WT macrophages were treated with MG132 (10 μM) and TNF-α (1000 U/ml) or TNF-α+zVAD (50 μM) for various time intervals. Lysate were collected and incubated with TUBEs for 16 hours at 4°C as described in experimental methods. Immuno-precipitates (C) and lysates (D) were examined by western blotting. Each experiment was repeated thrice.

**Figure S5.** **LUBAC does not impact RipK1 degradation.** (A, B) Densitometric analysis of western blots shown in Fig. 6 I. (C, D, E) Densitometric analysis of western blots shown in Fig. 6 K, L and M respectively. (F, G) Bone marrow-derived macrophages were treated with LPS (100ng/ml) + zVAD (50 μM) with or without Gliotoxin (5 μM) for various time intervals. (F) Cell lysates were examined by western blotting for RipK1 and RipK3 expression. (G) Cell viability was examined at 24 h by MTT assay. Graphs show the percentage of viable cells ± SEM relative to cells treated with LPS in the absence of zVAD. Each experiment was repeated thrice with triplicate samples.

**Figure S6.** **RipK1 degradation correlates with deceleration of cell death.** (A, B) WT macrophages were treated with LPS+zVAD. At various time intervals, cell viability and RipK1 levels were evaluated by MTT and western blotting respectively. Densitometric analysis of RipK1 and fold change in cell viability relative to controls was evaluated (B). Experiment was repeated thrice.
