# Supplementary figures and images for: Triad3a induces the degradation of early necrosome to limit RipK1-dependent cytokine production and necroptosis
Source: Cell Death Dis. 2018 May 22;9(6):592. doi: 10.1038/s41419-018-0672-0 (PMC5964080; doi:10.1038/s41419-018-0672-0)

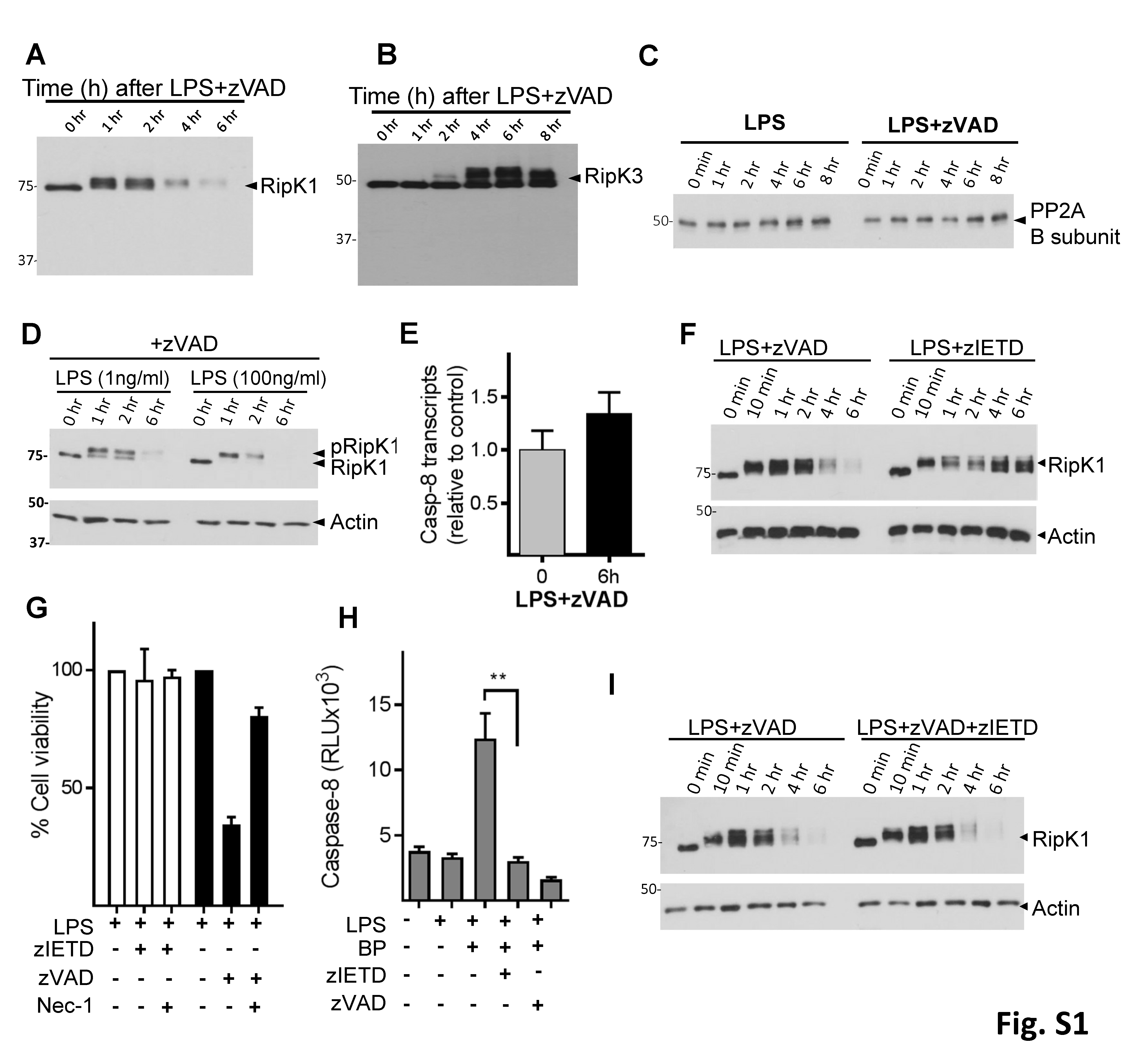

Supplement: Supplementary file 2 — Supplemental Figure 1 [file 41419_2018_672_MOESM2_ESM.tif]

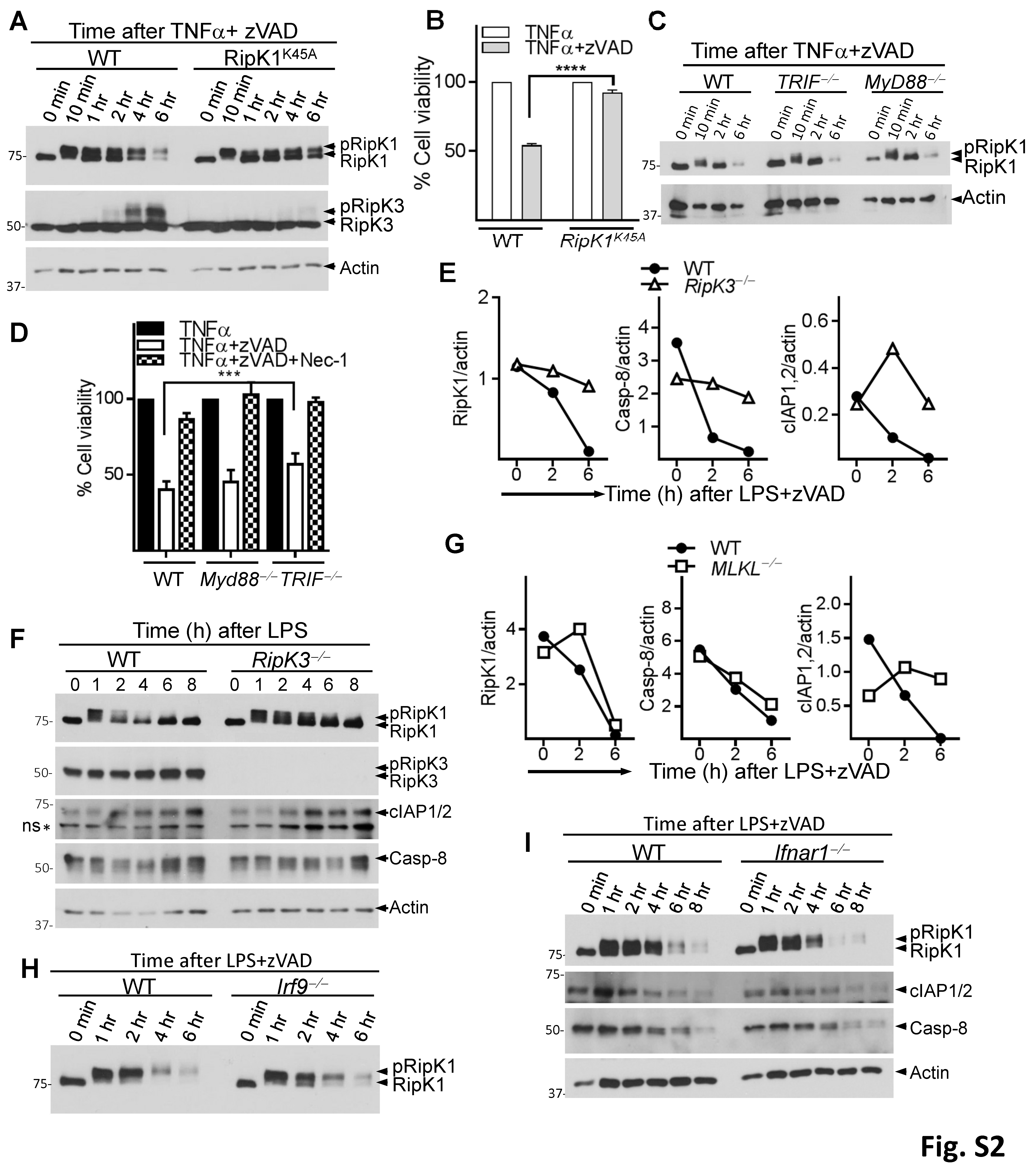

Supplement: Supplementary file 3 — Supplemental Figure 2 [file 41419_2018_672_MOESM3_ESM.tif]

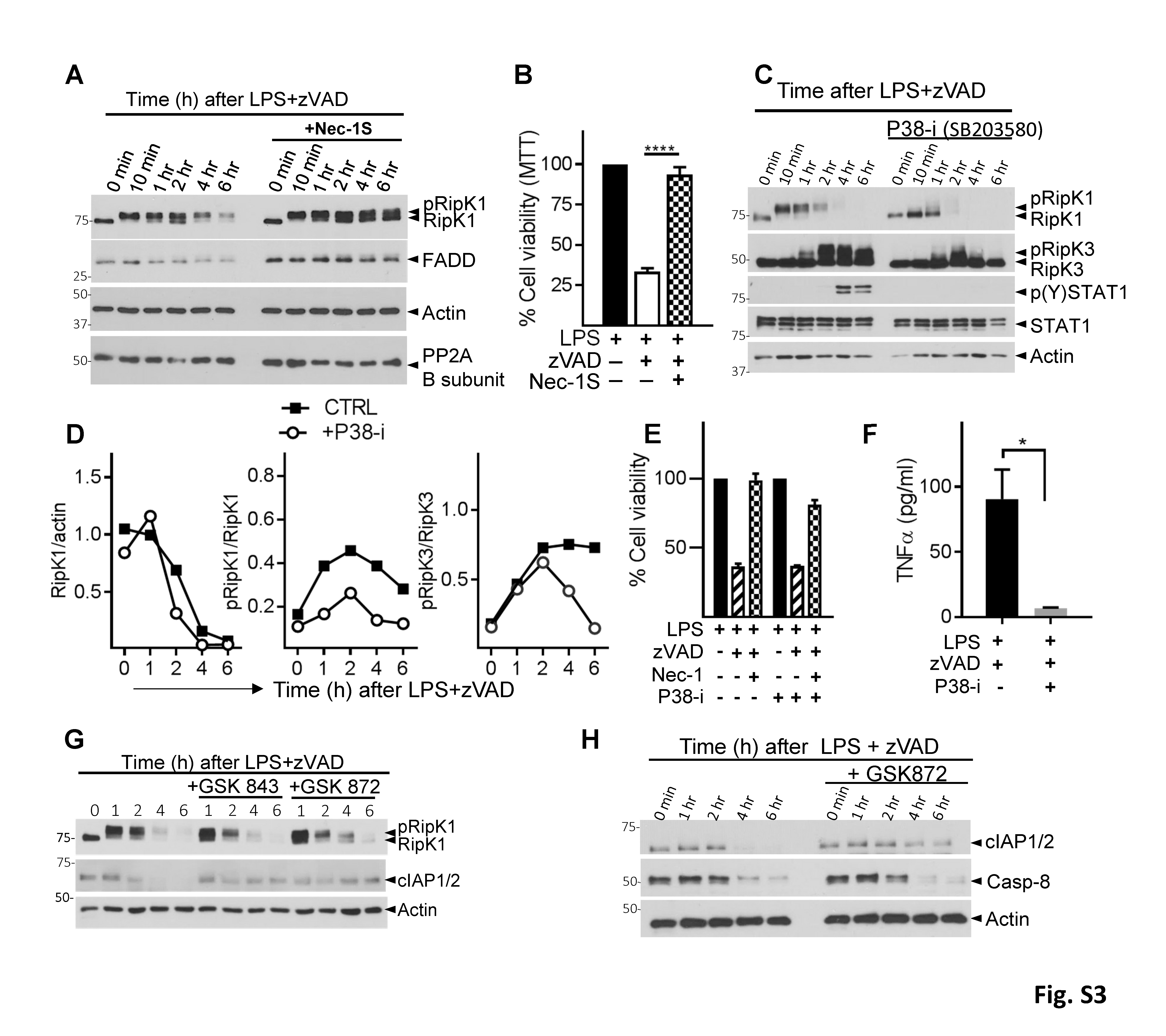

Supplement: Supplementary file 4 — Supplemental Figure 3 [file 41419_2018_672_MOESM4_ESM.tif]

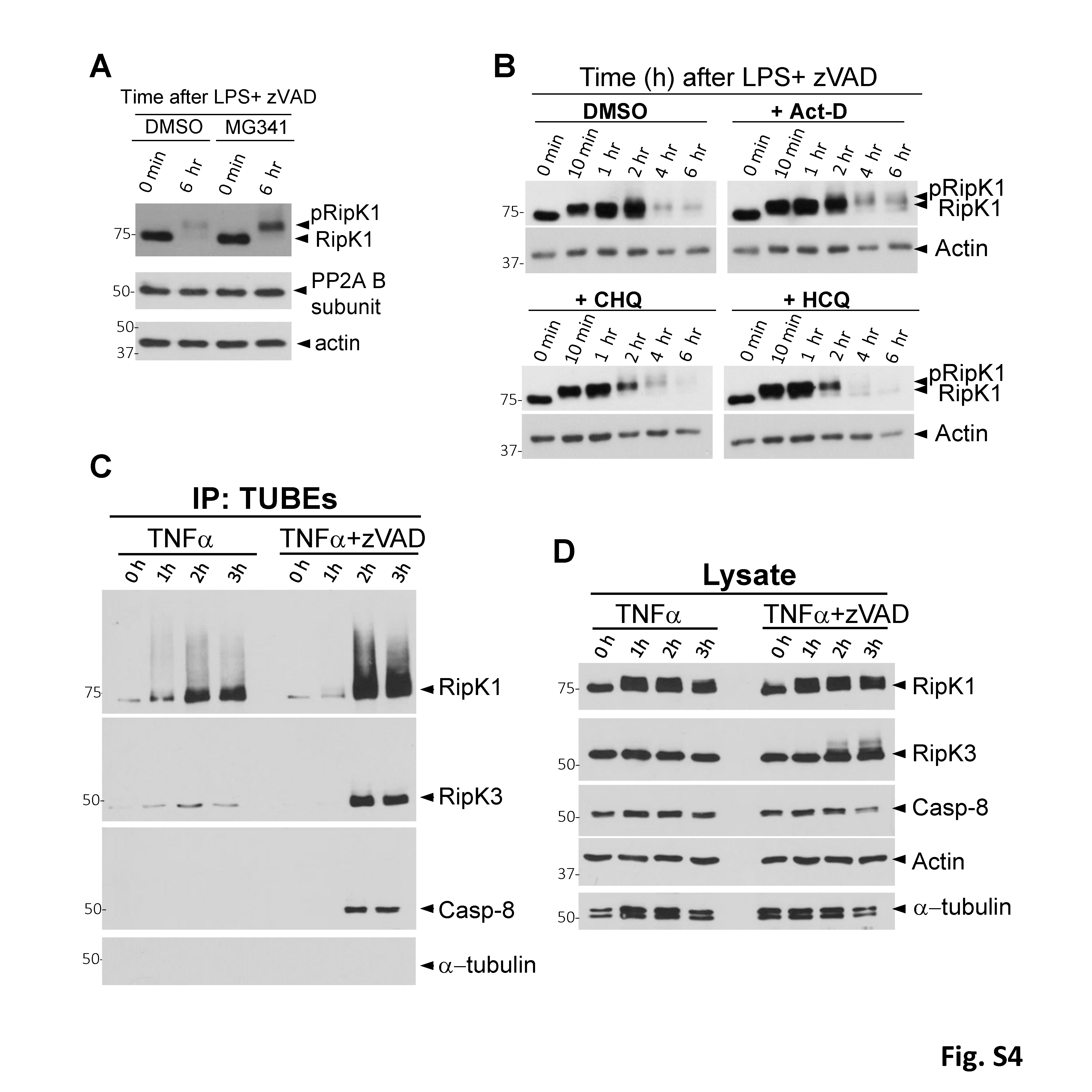

Supplement: Supplementary file 5 — Supplemental Figure 4 [file 41419_2018_672_MOESM5_ESM.tif]

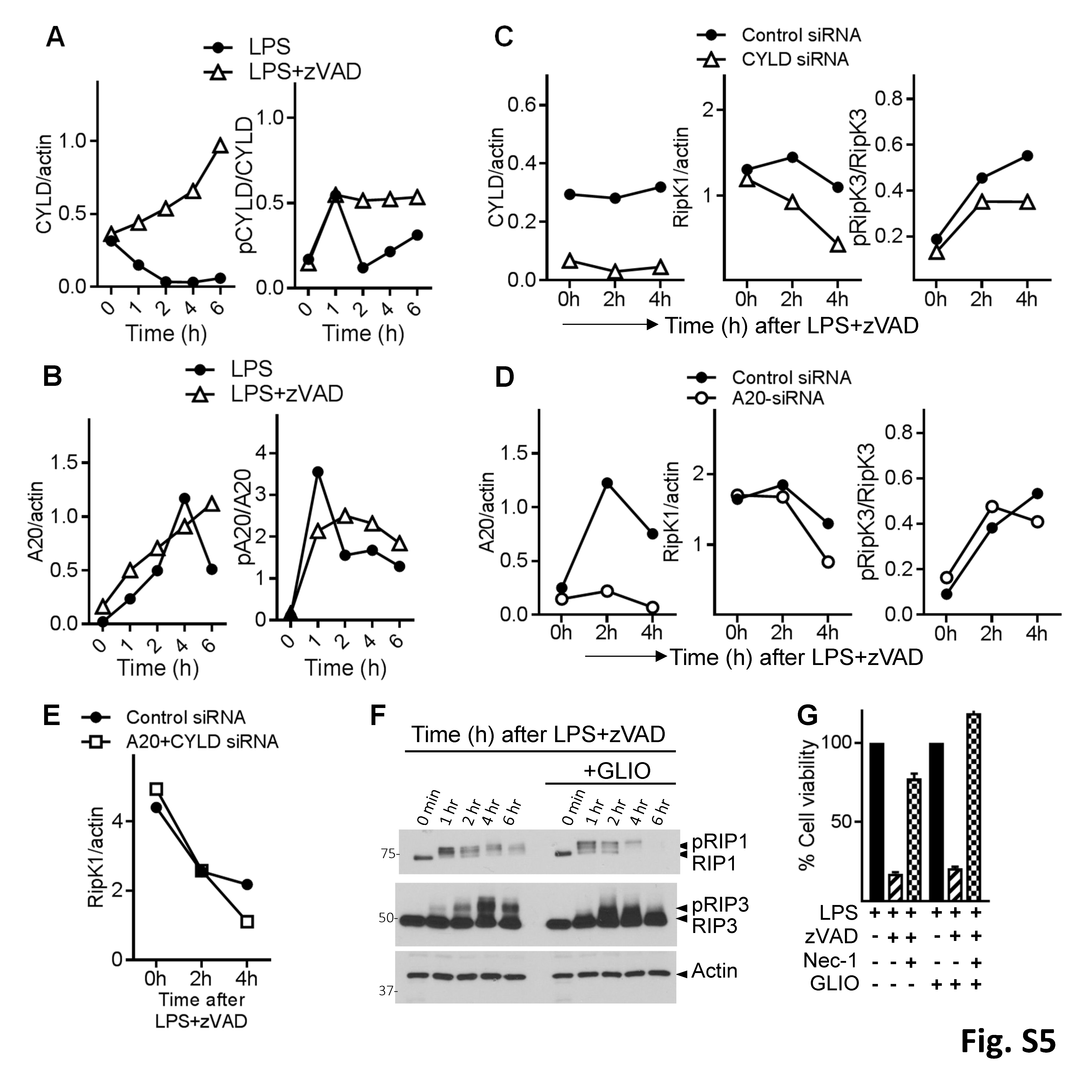

Supplement: Supplementary file 6 — Supplemental Figure 5 [file 41419_2018_672_MOESM6_ESM.tif]

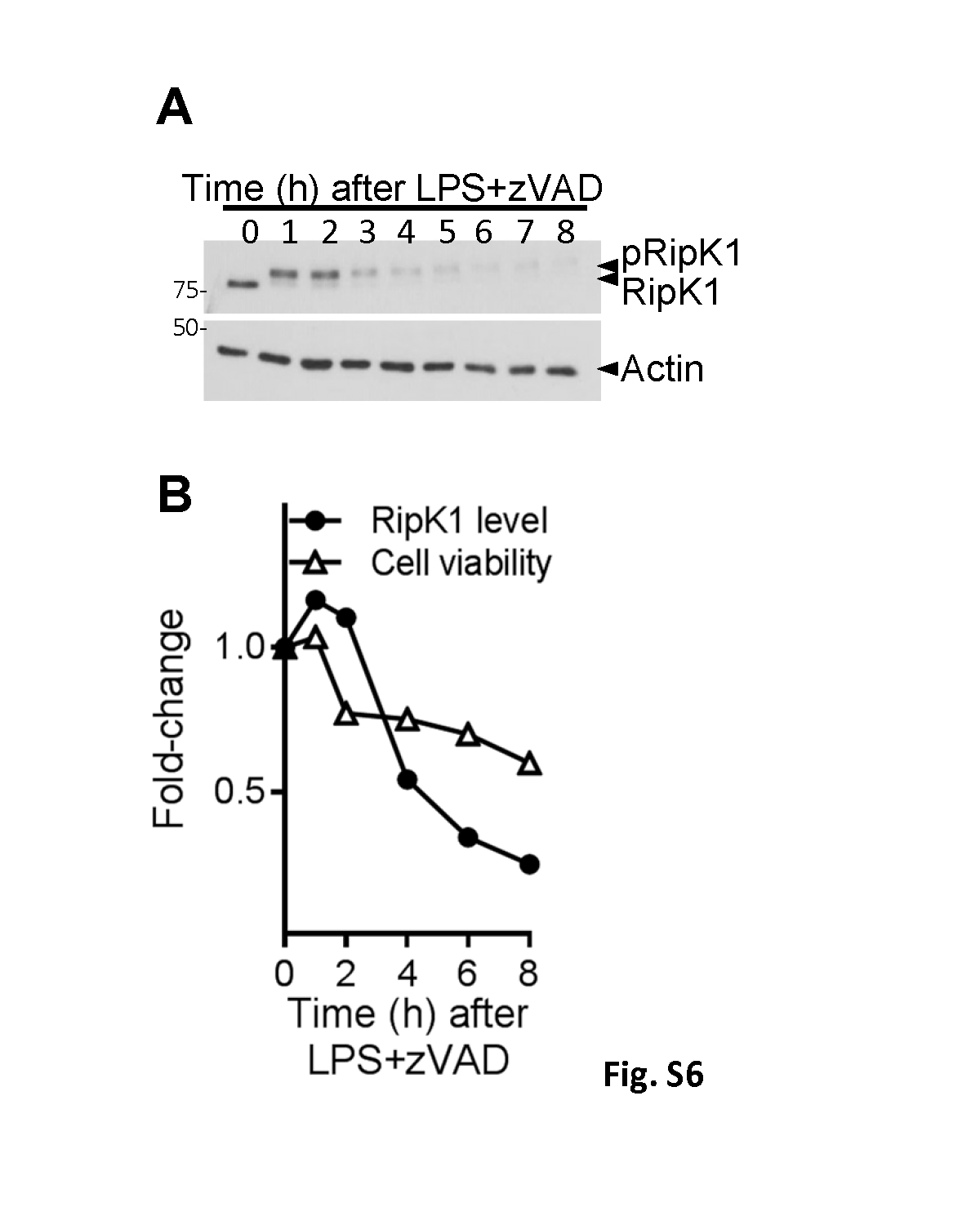

Supplement: Supplementary file 7 — Supplemental Figure 6 [file 41419_2018_672_MOESM7_ESM.tif]
