# Supplementary material for: Triad3a induces the degradation of early necrosome to limit RipK1-dependent cytokine production and necroptosis
Source: Cell Death Dis. 2018 May 22;9(6):592. doi: 10.1038/s41419-018-0672-0 (PMC5964080; doi:10.1038/s41419-018-0672-0)
Supplement: Supplementary file 8 — Supplemental data siRNA sequences [file 41419_2018_672_MOESM8_ESM.docx]

**L-055575-01-0005, ON-TARGETplus Mouse Cyld (74256) siRNA – SMARTpool**

A: ON-TARGETplus SMARTpool siRNA J-055575-09, Cyld

Target Sequence: UGAAAUGACUGAGCGAUAA

B: ON-TARGETplus SMARTpool siRNA J-055575-10, Cyld

Target Sequence: AGAAACAGACUCCGACUAA

C: ON-TARGETplus SMARTpool siRNA J-055575-11, Cyld

Target Sequence: CUGCAUUGAUGAUACGAUA

D: ON-TARGETplus SMARTpool siRNA J-055575-12, Cyld

Target Sequence: GGACAGAGAUAGUCAAUCC

**L-058907-02-0005, ON-TARGETplus Mouse Tnfaip3 (A20) (21929) siRNA – SMARTpool**

A: ON-TARGETplus SMARTpool siRNA J-058907-09, Tnfaip3 (A20)

Target Sequence: GCACCUAAGCCAACGAGUA

B: ON-TARGETplus SMARTpool siRNA J-058907-11, Tnfaip3 (A20)

Target Sequence: GCUGUGAAGAUACGAGAGA

C: ON-TARGETplus SMARTpool siRNA J-058907-12, Tnfaip3 (A20)

Target Sequence: AGAGACAUGCCUCGAACUA

D: ON-TARGETplus SMARTpool siRNA J-058907-17, Tnfaip3 (A20)

Target Sequence: GCUCAACUGGUGUCGUGAA

**ON-TARGET plus Mouse Rnf216 siRNA SMART pool:**

A-ON-TARGETplus SMARTpool siRNA J065113-05-RNF216 (Triad3a)

Target sequences: GCAGACAGCAGACGAUAUU

B- ON-TARGETplus SMARTpool siRNA J065113-06-RNF216 (Triad3a)

Target sequences: GAGCAGAAAGUCAUUAUAU

C- ON-TARGETplus SMARTpool siRNA J065113-07-RNF216 (Triad3a)

Target sequences: AGAGAAGACCGGCUUAUUA

D- ON-TARGETplus SMARTpool siRNA J065113-08-RNF216 (Triad3a)

Target sequences: ACAGAGCUCUUAUCAAAUC

**ON-Target plus Non-targeting pool**

Catalog Item

D-001810-10-20
